# Functional assessment of hydrophilic domains of late embryogenesis abundant proteins from distant organisms

**DOI:** 10.1111/1751-7915.13416

**Published:** 2019-04-22

**Authors:** Yingying Liu, Heng Zhang, Jiahui Han, Shijie Jiang, Xiuxiu Geng, Dong Xue, Yun Chen, Chen Zhang, Zhengfu Zhou, Wei Zhang, Ming Chen, Min Lin, Jin Wang

**Affiliations:** ^1^ Biotechnology Research Institute Chinese Academy of Agricultural Sciences Beijing 100081 China; ^2^ College of Life Science and Engineering Southwest University of Science and Technology Mianyang 621000 China

## Abstract

Late embryogenesis abundant (LEA) proteins play a protective role during desiccation and oxidation stresses. LEA3 proteins are a major group characterized by a hydrophilic domain (HD) with a highly conserved repeating 11‐amino acid motif. We compared four different HD orthologs from distant organisms: (i) DrHD from the extremophilic bacterium *Deinococcus radiodurans*; (ii) CeHD from the nematode *Caenorhabditis elegans*; (iii) YlHD from the yeast *Yarrowia lipolytica*; and (iv) BnHD from the plant *Brassica napus*. Circular dichroism spectroscopy showed that all four HDs were intrinsically disordered in phosphate buffer and then folded into α‐helical structures with the addition of glycerol or trifluoroethanol. Heterologous HD expression conferred enhanced desiccation and oxidation tolerance to *Escherichia coli*. These four HDs protected the enzymatic activities of lactate dehydrogenase (LDH) by preventing its aggregation under desiccation stress. The HDs also interacted with LDH, which was intensified by the addition of hydrogen peroxide (H_2_O_2_), suggesting a protective role in a chaperone‐like manner. Based on these results, the HDs of LEA3 proteins show promise as protectants for desiccation and oxidation stresses, especially DrHD, which is a potential ideal stress‐response element that can be applied in synthetic biology due to its extraordinary protection and stress resistance ability.

## Introduction

Both desiccation and oxidation are widely studied extreme processes that are detrimental to the growth of organisms because they induce protein aggregation and nucleic acid damage (Rajpurohit and Misra, 2013; Atashgahi *et al*., 2018). Moreover, recent advancements have shown that the organismal responses to both stresses are similar, indicating that the mechanisms for protecting crucial biomolecules from damage are related (Fredrickson *et al*., 2008; Chakrabortee *et al*., 2010). Long‐term investigations into the molecular mechanism of desiccation resistance emphasized late embryogenesis abundant (LEA) proteins, which account for more than 4% of cellular proteins in cotton orthodox seeds (Hughes and Galau, 1989; Roberts *et al*., 1993; Shimizu *et al*., 2010). Furthermore, many reports have revealed that the heterologous expression of LEA proteins can significantly improve recipient oxidation resistance (Liu and Zheng, 2005; Kovacs *et al*., 2008; Shih *et al*., 2012; Sasaki *et al*., 2014; Wang *et al*., 2017).

Late embryogenesis abundant proteins were first characterized in cotton seeds three decades ago and are hydrophilic (Dure and Galau, 1981); their accumulation simultaneously occurs alongside seed maturation, desiccation and other forms of (a) biotic stresses (Chakrabortee *et al*., 2010; Hatanaka *et al*., 2013; Liu *et al*., 2013; Leeuwen *et al*., 2016; Bremer *et al*., 2017). Continuous engagement in research on LEA proteins has shown us that these family proteins are not restricted to plants, as they have also been documented in bacteria, fungi and animals (Hand *et al*., 2011). Based on the amino acid information and conserved motifs, LEA proteins are classified into at least seven distinct groups (Battaglia *et al*., 2008). Among them, group 3 LEA (G3LEA) proteins have been extensively studied in vegetative plants, revealing the important roles of these proteins in inducing and maintaining a temporary period of ‘anhydrobiosis’ or ‘cryptobiosis’ (Ried and Walker‐Simmons, 1993). Molecular shield activity or chaperone‐like activity was assumed as the typical function of LEA group 3 as they probably form physical electrostatic barrier, which thus reduces desiccation‐induced aggregation, and then the crucial enzymes, like lactate dehydrogenase (LDH), were protected (Chakrabortee *et al*., 2010, 2012; Olvera‐Carrillo *et al*., 2011; Kovacs and Tompa, 2012).

Loosely conserved 11‐mer amino acid units (motifs) are the main feature of G3LEA proteins and were previously identified as ‘TAQAAKEKAGE’ (Ried and Walker‐Simmons, 1993). The number and size of motifs in G3LEA proteins vary by species (Dure and Galau, 1981; Dure, 1993; Dure, 2001; Swire‐Clark and Marcotte, 1999). Structural exploration of G3LEA proteins using circular dichroism (CD) indicated that they are randomly coiled in aqueous solution but adopt a largely α‐helical conformation in the presence of glycerol or after desiccation, which is considered to be the prerequisite for LEA‐macromolecule interactions for protection under stresses (Tolleter *et al*., 2007). Although many studies implicated that the hydrophilic regions of LEA proteins were crucial to achieve their structural transformation that contributes to the organisms’ stress tolerance acquisition (Haaning *et al*., 2008; Rajpurohit and Misra, 2013; Cuevas‐Velazquez *et al*., 2016), details of the function of this hydrophilic region remain unclear. Additionally, previous work highlighted the importance of G3LEA protein motifs from soybean that contribute to their enhanced salt resistance (Liu and Zheng, 2005). Unfortunately, their work focused on the role of only 22‐mer motifs, which are not the common feature of G3LEA proteins. Therefore, investigating the role of 11‐mer motifs became necessary to get a better and whole‐scale understanding of G3LEA proteins. Herein, the 11‐mer motifs of G3LEA proteins are artificially labelled the hydrophilic domain (HD).

Regarding the exploration of the mechanism of living cells adapting to extreme conditions, *Deinococcus radiodurans* was selected as the research model due to its extraordinary stress resistance, especially under desiccation and oxidation (Makarova *et al*., 2001; Daly, 2009; Slade and Radman, 2011; Ujaoney *et al*., 2017). Coincidently, a gene named *dr_1172* has been characterized to encode a G3LEA protein bearing distinct 11‐mer repeating motifs, and its disruption leads to a 75% growth loss in desiccated cultures (Battista *et al*., 2001). However, little information provided from previous *dr_1172* study, including its functional domain identification and physiological activity. Herein, the protein encoded by *dr_1172* is designated DrG3LEA (*D* *einococcus* *r* *adiodurans* group 3 LEA protein). Henceforth, DrG3LEA, the representative from *D. radiodurans*, together with other three G3LEA proteins from *Caenorhabditis elegans* (animal), *Yarrowia lipolytica* (fungus) and *Brassica napus* (plant) was selected due to their sequence similarity, comparatively conserved HDs and their representativeness. More importantly, comparisons among these four HDs in structure, function and mechanism would be useful to generalize the ubiquitous properties of G3LEA proteins as well as for screening the anti‐stress element for future application in synthetic biology rather than employing varied full‐length LEA proteins.

In the present work, we characterized four new HDs from G3LEA proteins using bioinformatic analysis and CD analysis. Heterologous expression further identified that their functionality involves desiccation and oxidation tolerance enhancement of *E. coli,* and their protective functions were well‐elucidated according to their anti‐aggregation and stress‐strengthen interaction ability, in which they were shown to act in the same chaperone‐like manner as the G3LEA proteins behaved.

## Results

### *In silico* analysis of DrHD, CeHD, YlHD and BnHD

The hydrophilic domain (HD) of DrG3LEA (NP_294896) from *D. radiodurans* is named DrHD and contains 8 motifs, and hydrophilic residues account for 62.4% of the HD amino acids, whereas the HD of the group 3 LEA (G3LEA) protein (BAB88877) from *Brassica napus* exhibits the highest hydrophilicity (70.1%), is named BnHD and bears six motifs. The HD of the G3LEA protein (SEI35173.1) from *Y. lipolytica*, designated YlHD, has the highest number of motifs (27), and hydrophilic residues account for 57.9% of the HD amino acids. The HD from the *C. elegans* G3LEA protein (NP_001256171), named CeHD, has 24 motifs, in which the hydrophilic residues occupy 60.4% of the HD amino acids (Table 1). Obviously, the motif numbers varied by species, and the motif residues were not conserved among HDs as shown in Fig. S2. According to their hydrophilicity analysis shown in Kyte and Doolittle hydropathy plots (Fig. S1) (Kyte and Doolittle, 1982), these HDs were predominantly hydrophilic with few hydrophobic amino acid residues, which indicates their potential to form α‐helix secondary structures (Dure, 1993).

**Table 1:** Comparisons among four LEA proteins based on their amino acids (aa). These four LEA proteins were representative of those in bacteria (*D. radiodurans*), fungi (*Y. lipolytica*), plants (*B. napus*) and animals (*C. elegans*). The HDs were named DrHD, YlHD, BnHD and CeHD respectively

Accession No. in GenBank	Species	Amino acids of full length(aa)	Name of hydrophilic domain	Amino acids of domain(aa)	Molecular weight of domain(Da)	Numbers of 11‐mer motif	Hydrophilic residues percentage of domain (%)
NP_294896	*Deinococcus radiodurans* R1	298	DrHD	142 (104–245)*	14707	8	62.4
NP_001256171	*Caenorhabditis elegans*	733	CeHD	477 (226–702)*	50138.6	24	60.4
SEI35173.1	*Yarrowia lipolytica*	748	YlHD	575 (87–661)*	62262.7	27	57.9
BAB88877	*Brassica napus*	226	BnHD	121 (42–162)*	12995.5	6	70.1

The asterisk indicates the position from the starting aa to the terminating aa.

### Structural transformation of HDs from intrinsically disordered to ordered with the addition of glycerol or trifluoroethanol (TFE)

Studies have shown that most late embryogenesis abundant (LEA) proteins are intrinsically disordered proteins (IDPs) under natural conditions that are transformed into ordered structures in dry environments (Tolleter *et al*., 2007; Chakrabortee *et al*., 2012; Hundertmark *et al*., 2012; Hatanaka *et al*., 2013; Cuevas‐Velazquez *et al*., 2016). All four HDs, DrHD, CeHD, BnHD and YlHD, were predicted to be intrinsically disordered using Cspritz and IUPred (data not shown).

**Figure 1:**
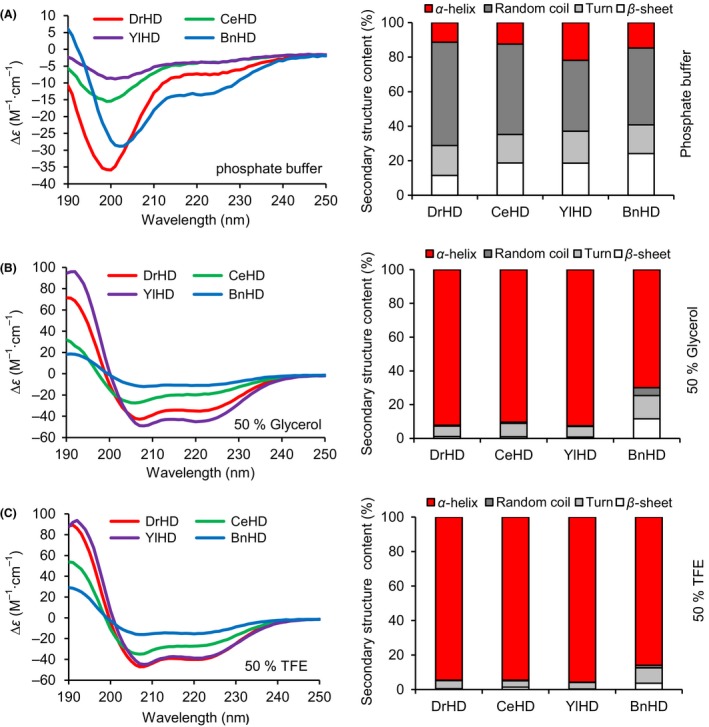
CD analysis of HDs. The structural transformation of HDs was promoted by the water‐deficient reagents 50% glycerol (B) and 50% TFE (C) compared to phosphate buffer (A). The secondary structure composition was estimated from CD spectra. Percentages of random coil (dark grey), turn (light grey), beta‐sheet (white) and α‐helix (red) are deduced from CD spectra using the CDPro program.

Henceforth, to investigate these four HD polypeptides’ secondary structure in aqueous solution, their genes were cloned into the expression vector pET28a with a 6xHis tag. The expressing strains (BL21) were induced by IPTG (0.1 mM), and the produced proteins were purified by affinity chromatography. The protein purity reached 90% after gel filtration (Fig. S3), and the molecular mass of the purified DrHD, CeHD, YlHD and BnHD proteins was approximately 14.7 kDa, 50.1 kDa, 62.3 kDa and 13 kDa respectively. However, according to the SDS‐PAGE results, the HDs migrated to a higher molecular mass due to their hydrophilic character, which is often used to identify IDPs (Tompa, 2002; Chakrabortee *et al*., 2010). The proteins were analysed by far‐UV circular dichroism (CD) spectroscopy. The spectra of all four HDs had a negative peak at 198 nm in phosphate buffer, indicating that DrHD, CeHD, BnHD and YlHD are natively unstructured in solution (Fig. 1A). As shown by the stacked bar graphs in the right half of Fig. 1, these HDs are folded and formed α‐helices with the addition of glycerol (50%) (generally used to mimic water‐loss environment) or TFE (50%) (trifluoroethanol, a well‐known α‐helix inducer) (Cuevas‐Velazquez *et al*., 2016), with a high ellipticity at 192 nm and minima at 208 and 220 nm (Fig. 1B and C). In addition, DrHD, YlHD and CeHD are able to gain higher levels of helicity than BnHD (Table S2), implying that, on one hand, these four HDs are the core domain for determining their function, as previous work demonstrated that folding into α‐helix is required for activating their protective function under water‐deficient environments (Cuevas‐Velazquez *et al*., 2016); on the other hand, different α‐helix levels of HDs might lead to differences in their physiological function.

### Hydrophilic domains (HDs) confer improved desiccation and oxidation resistance to *E. coli*

**Figure 2:**
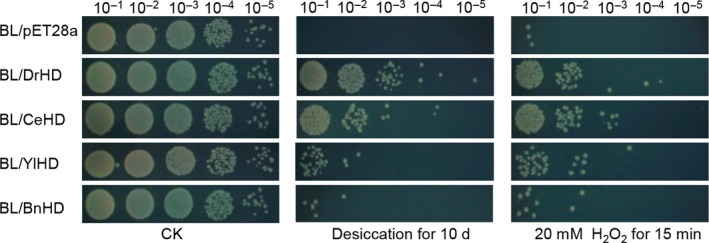
Survival phenotype plate assay of *E. coli* recombinant strains under desiccation and oxidation. BL/DrHD, BL/CeHD, BL/YlHD and BL/BnHD are the expressing strains, and BL/pET28a recombinant carried an empty pET28a vector. Serial 10‐fold dilutions of OD‐standardized recombinant strains (OD ≈ 0.6) were spotted onto LB plates (6 μl) after desiccation for 10 days and 20 mM H_2_O_2_ treatment for 15 min; CK stands for untreated culture control.

To explore the function of HDs against desiccation and oxidation, four *E. coli* transformants harbouring HD fragments were employed, and transformants harbouring pET28a served as the control. Clearly, compared to the control, the four transformants exhibited enhanced desiccation resistance after 10 days of treatment. In particular, BL/DrHD displayed the strongest viability, approximately one order of magnitude higher than that of BL/CeHD (Fig. 2). Similarly, BL/DrHD or BL/CeHD exhibited improved oxidation tolerance that was approximately one order of magnitude stronger than those of BL/YlHD and BL/BnHD, which seemingly conform to the same trend as desiccation. With regard to oxidation resistance, further analysis revealed that both BL/DrHD and BL/CeHD transformants, compared to BL/YlHD and BL/BnHD, displayed minimum loss of antioxidant activity, as shown in Fig. S4. Taken together, these results not only indicated the capability of HDs to enhance desiccation and oxidation tolerance of *E. coli* but also proved that the level of α‐helix formation in G3LEA proteins tends to impact their physiological function, increased the α‐helix level, and improve stress resistance, combining with the secondary structure measurements (Fig. 1 and Table S2).

### HDs reduce desiccation‐induced enzyme aggregation and inactivation

**Figure 3:**
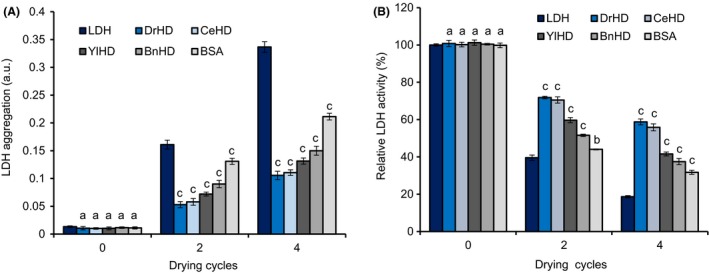
Effect of desiccation on LDH aggregation and activity. LDH aggregation (A) and (B) activity on repeated desiccation. The molar ratio of LDH and tested proteins was 1:3 on aggregation assay, while at 1:2.5 molar ratio on enzyme activity measurement. These measurements were performed three times for each case, and their *P*‐values were calculated based on Tukey multiple comparisons using by R statistics. The letters ‘a’, ‘b’ and ‘c’ represent ‘not significantly different (*P *>* *0.05)’, ‘significantly different (0.001 < *P *<* *0.01)’ and ‘extremely significantly different (*P *<* *0.001)’, respectively, compared to LDH alone.

These HDs from LEA proteins were highly hydrophilic and disordered, which were known to reduce desiccation‐induced enzyme aggregation, such as lactate dehydrogenase (LDH), which catalyses the conversion of lactate to pyruvic acid (Goyal *et al*., 2005; Chakrabortee *et al*., 2007; Liu *et al*., 2013). Therefore, we speculated that these HDs might perform stabilizing activity *in vitro* on LDH that was commonly used for aggregation and enzymatic activity protection assay (Reyes *et al*., 2005), as well as bovine serum albumin (BSA) serving as the positive control. It was clear to see that LDH marked aggregation subjected to cycles of desiccation (Fig. 3A), addition of HDs can significantly decrease the aggregation of LDH compared to BSA mixture and negative control (only LDH), respectively, even after four cycles of desiccation, suggesting their aggregation‐preventing ability. Noticeably, DrHD displayed the better job inhibiting the aggregation of LDH. The enzymatic activity of LDH was impaired under the dry conditions, especially after four cycles, and < 20% of the undried LDH activity was retained. Conspicuously, the loss of LDH enzymatic activity was significantly neutralized with the additional HDs and BSA. DrHD and CeHD performed excellently, preserving up to 60% of the LDH activity, and even BnHD protected up to 40% of the activity, which was slightly higher than the 38% protected by BSA (Fig. 3B). The HD protection of LDH enzymatic activity under oxidation stress followed a trend similar to that in desiccation (Fig. S5). DrHD and CeHD clearly exhibited the strongest protection ability (over 65% of LDH activity retained) of the HDs.

### HDs can interact with lactate dehydrogenase (LDH)

**Figure 4:**
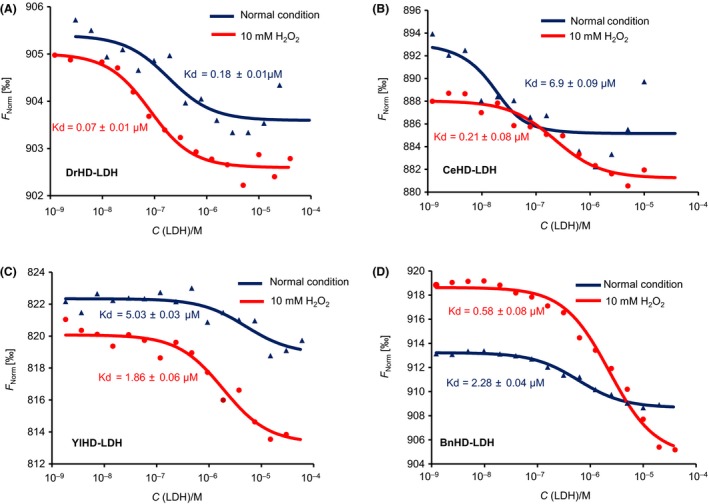
Interaction of HDs with LDH. A protein–protein interaction method, microscale electrophoresis, was employed to characterize the relationship between LDH and DrHD (A), CeHD (B), YlHD (C) and BnHD (D). The Kd value represents the dissociation constant indicating their affinity. The binding curve under normal conditions (phosphate buffer) is shown in dark blue, and the binding curve under 10 mM H_2_O_2_ treatment is shown in red (phosphate buffer supplemented with 10 mM H_2_O_2_).

Many research articles have reported that the enzymatic activity protection of G3LEA proteins is achieved due to their binding of the target molecules and then enveloping them and allowing them to avoid direct exposure to harsh conditions (Chakrabortee *et al*., 2010; Hundertmark *et al*., 2011; Sasaki *et al*., 2014). Thus, a protein–protein interaction assay was introduced to explore the binding ability of HDs. With regard to the dissociation constant (Kd), which represents the affinity of the binding participants, the interaction was strengthened with decreasing Kd value. Accordingly, the protection levels of HDs were deliberately demonstrated. As the microscale thermophoresis results are shown in Fig. 4, all four HDs can bind LDH under normal conditions, and the interaction became strengthened with the addition of 10 mM hydrogen peroxide, which not only revealed their chaperone‐like function but also dynamically disclosed the complex configurational change. More importantly, binding curves illustrated that the DrHD/LDH pair displayed the strongest interaction, with a lower *K*_d_ value (*K*_d_ = 0.18 ± 0.01 μM) than CeHD (6.9 ± 0.09 μM), YlHD (5.03 ± 0.03 μM) and BnHD (2.28 ± 0.04 μM) under normal conditions, suggesting that DrHD had the strongest protein binding ability and that their binding was enhanced in the presence of 10 mM H_2_O_2_ (Fig. 4A). To some extent, the close complex formation between HDs and LDH under oxidation conditions reflected their structural flexibility.

## Discussion

Extensive studies focusing on an organism's adaptation against harsh environments have highlighted the importance of late embryogenesis abundant (LEA) proteins found in many species ranging from plants and animals to microbes as they were highly induced in dry environments (Tompa, 2002; Gal *et al*., 2004; Tolleter *et al*., 2007; Chakrabortee *et al*., 2010; Hand *et al*., 2011). The accumulated LEA proteins act in a chaperone‐like manner to protect the organisms from various stresses, for example, salt, desiccation and oxidation (Hoekstra *et al*., 2001; Shimizu *et al*., 2010; Cuevas‐Velazquez *et al*., 2016; Wang *et al*., 2017). Further bioinformatic and structural analyses revealed that 11‐mer motifs specific to group 3 LEA (G3LEA) proteins (HD) might be the crucial regions determining their whole‐length functions (Dure, 1993; Xue *et al*., 2011). Unfortunately, detailed research focusing on this hydrophilic region, such as the study of its physiological function, structural transformation and protective mechanism, has rarely been reported. The annotation of the genome of *D. radiodurans*, the world's most tenacious extremophile model against radiation, desiccation and oxidation (Chen *et al*., 2017; Jeong *et al*., 2017; Schmier *et al*., 2017; Ujaoney *et al*., 2017; Zhou *et al*., 2017), indicated that there were three putative LEA homologous proteins (Makarova *et al*., 2001), in which a gene named *dr_1172* bearing classic 11‐mer repeating motifs (hydrophilic domain, HD) was functionally characterized and responsible for the strain's desiccation resistance (Battista *et al*., 2001). Therefore, HD from *D. radiodurans* together with other three sequence‐similar HDs, CeHD, BnHD and YlHD, representing animal, plant and fungus, respectively, was investigated in this study.

Protein sequence analysis revealed that the motifs were conserved among these four HDs (Table 1), which to some extent indicated their structural and functional similarity. *In silico* analysis revealed that these four HDs exhibited disordered structures that were abundant with random coils, which was verified by using circular dichroism (CD) spectrometry in phosphate buffer (Fig. 1). Due to their high proportion of hydrophilic amino acid residues, these LEA proteins were mainly converted from disordered status into regular α‐helices under desiccation condition (Hand *et al*., 2011) such as the presence of glycerol or TFE, which are commonly used reagents used to characterize α‐helix formation potential (Cuevas‐Velazquez *et al*., 2016). Structural identification showed the potential of these HDs to form α‐helices, which was in agreement with the LEA structural features (Hand *et al*., 2011), implicating that the decisive role of HD holding the whole‐length structure conversion under water‐loss environments. More interestingly, the α‐helix formation level varied and might be correlated with their physiological function, as compared to its counterparts shown in Fig. 1 and Fig. 2, and BnHD performed slight desiccation and oxidation enhancement with the least α‐helix formation. The high configurational flexibility of intrinsically disordered proteins (IDPs), including LEA, was identified to function in a chaperone‐like manner by preventing the protein's aggregation and protecting the enzymes’ activity (Chakrabortee *et al*., 2007, 2010; Tolleter *et al*., 2007). Apparently, all four of these HDs reduced desiccation‐ and oxidation‐induced enzyme aggregation and inactivation (Fig. 3 and Fig. S5), from which their important function similar to intensively studied chaperones, such as HSPs, was indicated (Dure, 1993; Kovacs and Tompa, 2012; Salleh *et al*., 2012; Hatanaka *et al*., 2013). But Intrinsically, the protective role of HD, same with LEA proteins that had been previously termed as a molecular shield or chaperone‐like activity (Chakrabortee *et al*., 2010, 2012; Kovacs and Tompa, 2012), was distinct from identified canonical chaperones. In structure, these four HDs same to are naturally in an unstructured state, while the classic HSPs are well‐structured with just a small disordered segment (Kovacs and Tompa, 2012). As for mechanism, these HSPs were assembled together to form a cooperative machinery undergoing protein recognition, disaggregation and ATP‐driving refold processes (Chakrabortee *et al*., 2007; Hand *et al*., 2011), whereas our HD studies revealed that they can weakly bind to enzymes such as LDH as indicated in Fig. 4, which is similar to a previous study explained as steric interference (Chakrabortee *et al*., 2012; Kovacs and Tompa, 2012), and intriguingly, their interaction was strengthened with addition of H_2_O_2_, which might be induced by the partially binding of HD with misfold to prevent further collisions (Kovacs and Tompa, 2012). Besides, HDs can also bind to MDH (data not shown) implicating that there are no specific binding sites, which is totally different with molecular chaperone, like HSP70, co‐immunoprecipitating with a polyQ‐containing protein (Chakrabortee *et al*., 2012). In addition, these HDs can inhibit LDH aggregation and protect its enzymatic activity without additional energy (Olvera‐Carrillo *et al*., 2011; Schopf *et al*., 2017) as well as without carbohydrates, such as trehalose (Kovacs and Tompa, 2012). Combining together, HD served as the molecular basis of G3LEA protection mechanism as shown in Fig. S6.

Interestingly, these HDs had *in vivo* antioxidant activity, which was seen as a moonlighting function supplementing their anti‐desiccation ability (Haaning *et al*., 2008). Certainly, the relationship between oxidation and desiccation was clearly demonstrated before the impact of desiccation was determined by the levels of biomolecule oxidation (Fredrickson *et al*., 2008). In other words, the fewer the oxidized metabolites produced, the higher the desiccation resistance. Apart from that, the similar effect trend under both conditions through heterologous expression and LDH enzymatic activity protection was noticeable. Oxidation can cause direct damage of proteins by destroying their three‐dimensional structure, leading to misfolding and aggregation, which is intrinsically different from the generated effect from glycerol or TFE that were used to identify LEA or IDPs potential forming α‐helix as well as used to stabilize the target proteins when exposed to harsh conditions (Corrêa and Farah, 2007; Cuevas‐Velazquez *et al*., 2016), and there was rarely reported that both of them were seen as damage‐caused reagents on biomolecules. Therefore, it was not difficult to understand that the addition of glycerol or TFE cannot cause the binding affinity change in LDH with HDs.

Obviously, CeHD and DrHD played an outstanding role in greatly increasing *E. coli* viability, preventing protein aggregation, minimizing the loss of LDH enzymatic activity and increasing binding ability. Compared to CeHD, DrHD is very advantageous for synthetic biology due to its small size and its equal or better efficacy. Further sequence analysis revealed that both DrHD and CeHD motifs were more conserved than those of the other two HDs (Fig. S2), especially key residues (Dure, 1993, 2001). Therefore, explorations of the crucial key residues would be meaningful to unravel the structural and functional basis of HDs. Our results implicated that these 11‐mer repeating motifs were responsible for the full‐length physiological function, providing guidance for future investigations as to whether their function is related to the motif numbers since the pioneering work indicative of the increased salt tolerance of duplicated motifs (Liu and Zheng, 2005).

In conclusion, we first characterized four putative HDs that were indispensable to G3LEA protein function undergoing structural transformation and providing chaperone‐like protection. These selected HDs enhanced *E. coli* desiccation and oxidation resistance, and furthermore, these HDs can inhibit desiccation‐induced aggregation and protect enzyme activity. Most importantly, the great performance of DrHD encouraged us to continue exploring its specificity and the hidden robustness of the G3LEA proteins, and inspired us to look for brief functional elements in future work for biosynthetic applications.

## Experimental procedures

### Sample acquisition and growth conditions

The bacterial strains and plasmids used in this study are described in Table 2. *E. coli* strains were cultured at 37°C in LB broth (1% tryptone, 0.5% yeast extract and 0.5% NaCl) or on LB plates with 50 μg ml^−1^ kanamycin or the appropriate antibiotic.

**Table 2:** Bacterial strains and plasmids used in this study

Strains/plasmids	Description	Source
Strains		
Trans10	Host for cloning vectors	TransGen Biotech
BL21	F^−^ *omp*T *hsd*S_B_ (r_B_^−^ m_B_^−^) *gal dcm* (DE3)	TransGen Biotech
BL/pET28a	The control strain harbouring the empty vector pET28a, Kan^r^	This study
BL/DrHD	The recombinant BL21 strain containing pET‐*DrHD*, Kan^r^	This study
BL/CeHD	The recombinant BL21 strain containing pET‐*CeHD*, Kan^r^	This study
BL/YlHD	The recombinant BL21 strain containing pET‐*YlHD*, Kan^r^	This study
BL/BnHD	The recombinant BL21 strain containing pET‐*BnHD*, Kan^r^	This study
Plasmid		
pET28a (+)	pBR322 and f1 ori, KmR, commercial vector for protein overexpression	Novagen
pET‐*DrHD*	pET28a‐derived plasmid carrying the *DrHD* gene with HD	This study
pET‐*CeHD*	pET28a‐derived plasmid carrying the *CeHD* gene with HD	This study
pET‐*YlHD*	pET28a‐derived plasmid carrying the *YlHD* gene with HD	This study
pET‐*BnHD*	pET28a‐derived plasmid carrying the *BnHD* gene with HD	This study

### Construction of heterologous expression system for DrHD, CeHD, YlHD and BnHD in *E**. **coli*

*DrHD* was amplified from the genomic DNA of *D. radiodurans* using specific primers. Similarly, the original nucleotide sequences of the *CeHD*,* YlHD* and *BnHD* genes were engineered and synthesized by BGI (Beijing, China). All forward primers contained a BamHI site (underlined), and the reverse primers contained a XhoI site (underlined). Four PCR products were cloned into a pET28a vector using the Seamless Assembly Cloning Kit (Cat# C5891‐25; Clone Smarter, Houston, Texas, USA). All recombinant plasmids (pET‐*DrHD*, pET‐*CeHD*, pET‐*YlHD*, pET‐*BnHD*) and an empty pET28a vector were introduced into *E. coli* host strain BL21 (DE3) to generate the transformed strains BL/DrHD, BL/CeHD, BL/YlHD, BL/BnHD and BL/pET28a (control). Primers were listed in Table S1.

### Circular dichroism (CD) spectroscopic analysis

Far‐UV circular dichroism (CD) spectra were generated using a Jasco‐720 spectropolarimeter (Jasco Instrument, Tokyo, Japan) with a 0.1 mm path length cuvette at wavelengths of 190‐250 nm as previously described in detail (Chakrabortee *et al*., 2010). Recombinant proteins were diluted to 0.2 mg ml^−1^ in 10 mM phosphate buffer (pH 7.0). Samples were placed into the spectrometer on a single window and scanned immediately. Relative secondary structure content of all proteins was analysed with CDPro software.

### Desiccation and oxidation stress tolerance in *E**. **coli*

*Escherichia coli* recombinant strain cultures were induced with IPTG at a final optimal concentration of 0.1 mM for 4 h, and the cells were desiccated and treated with H_2_O_2_ as previously described (Rajpurohit and Misra, 2013; Jiang *et al*., 2017; Wang *et al*., 2017). Different serial dilutions of these cells were plated onto LB agar plates and incubated at 37°C for 1 day before colonies were observed and counted. All experiments were performed three times. See Supporting Experimental procedures for more details.

### Lactate dehydrogenase (LDH) aggregation and activity assay *in vitro*

Lactate dehydrogenase (LDH) aggregation and activity was assayed as described previously with some modifications (Goyal *et al*., 2005; Hatanaka *et al*., 2013; Liu *et al*., 2013). For the aggregation measurement, 100 μl of 2 mg ml^−1^ LDH was mixed with or without purified recombinant proteins at molar ratio of 3:1 (test protein: LDH in an Eppendorf tube. And then samples were set in a speed‐vac VC‐960 for desiccation by centrifugal concentration under reduced pressure for 4 h. Once completely desiccated, these samples were rehydrated in the same volume of 10 mM phosphate buffer. Samples were then dried, and the rehydration cycle was repeated. Aggregation was assessed by measuring apparent absorbance at 340 nm. All assays were conducted in a total starting volume of 200 μl and were assayed in triplicate. For enzymatic activity measurement, LDH and additives were diluted in 25 mM Tris–HCl (pH 7.5) to a final concentration of 8.3 μg ml^−1^. For desiccation treatment, the enzyme mixture was left in a desiccator box for 24 h and then rehydrated in the same volume of 25 mM Tris–HCl (pH 7.5). LDH activity was monitored as the rate of absorbance change at 340 nm for 1 min due to the conversion of NADH to NAD^+^ at 25°C. The rate determined for the untreated samples was considered 100% in all graphs. See more details in Supporting Experimental procedures.

### Microscale thermophoresis (MST) measurements

Microscale thermophoresis (MST) experiments were performed as described previously (Zhan *et al*., 2016). The purified proteins (DrHD, CehD, YlHD and BnHD) were labelled with the Monolith NTTM Protein Labeling Kit RED‐NHS according to the supplied labelling protocol (Cat# L011; NanoTemper Technologies Co.,Ltd., Chaoyang, Beijing, China). Labelled proteins were used at a concentration of 2 μM. Label‐free LDH was used as the ligand. LDH was diluted from 71 μM in binding buffer (20 mM HEPES, 150 mM KCl, pH 7.4). For the oxidation treatment, H_2_O_2_ solution was added to the enzyme–protein mixture to obtain a final concentration of 10 mM. Ten microlitres of the LDH solution was mixed with 10 μl of labelled protein. After mixing, the samples were loaded on standard treated capillaries. The measurements were taken using a Monolith NT.115 (NanoTemper Technologies) that was operated at 20% light‐emitting diode (LED) and 60% MST power. Data analyses were performed using NanoTemper Analysis software v.1.2.101 (NanoTemper Technologies).

## Conflict of interest

None declared.

## Supporting information


**Fig. S1.** Hydropathic index plot of the HD amino acid sequences analyzed by using the Kyte‐Doolittle algorithm. Regions with a hydropathy score below zero are hydrophilic.
**Fig. S2.** Comparison of the repeating 11‐mer motif in HDs.
**Fig. S3.** SDS‐PAGE analysis of four purified HD proteins. Lane 1, purified BnHD; lane 2, purified DrHD; lane 3, purified CeHD; lane 4, purified YlHD; lane M, molecular weight marker (kDa).
**Fig. S4.** The total antioxidant activity of the transformants was detected by a spectrometer.
**Fig. S5.** The role of HDs in protecting LDH against H_2_O_2_ stress.
**Fig. S6.** Chaperone‐like function model of HD. HD can bind with native proteins, especially crucial enzymes in metabolic pathways, in a weak manner under normal conditions.
**Table S1.** List of primers used in this study.
**Table S2.** Secondary structure content in four HD proteins was obtained by far‐UV CD spectrometry and calculated with the CDPro program.
