# Supplementary material for: Functional assessment of hydrophilic domains of late embryogenesis abundant proteins from distant organisms
Source: Microb Biotechnol. 2019 Apr 22;12(4):752–62. doi: 10.1111/1751-7915.13416 (PMC6559209; doi:10.1111/1751-7915.13416)
Supplement: Supplementary file 1 — Fig. S1. Hydropathic index plot of the HD amino acid sequences analyzed by using the Kyte‐Doolittle algorithm. Regions with a hydropathy score below zero are hydrophilic. Fig. S2. Comparison of the repeating 11‐mer motif in HDs. Fig. S3. SDS‐PAGE analysis of four purified HD proteins. Lane 1, purified BnHD; lane 2, purified DrHD; lane 3, purified CeHD; lane 4, purified YlHD; lane M, molecular weight marker (kDa). Fig. S4. The total antioxidant activity of the transformants was detected by a spectrometer. Fig. S5. The role of HDs in protecting LDH against H2O2 stress. Fig. S6. Chaperone‐like function model of HD. HD can bind with native proteins, especially crucial enzymes in metabolic pathways, in a weak manner under normal conditions. Table S1. List of primers used in this study. Table S2. Secondary structure content in four HD proteins was obtained by far‐UV CD spectrometry and calculated with the CDPro program. [file MBT2-12-752-s001.docx]

Supporting Information

[Functional assessment of](https://www.ncbi.nlm.nih.gov/pubmed/25616417) hydrophilic domains of late embryogenesis abundant proteins from distant organisms

Yingying Liu,^1^ Heng Zhang,^1^ Jiahui Han,^1^ Shijie Jiang,^1,2^ Xiuxiu Geng,^1,2^ Dong Xue,^1^ Yun Chen,^1^ Chen Zhang,^1^ Zhengfu Zhou,^1^ Wei Zhang,^1^ Ming Chen,^1^ Min Lin,^1^ and Jin Wang^1,*^

^1^ *Biotechnology Research Institute, Chinese Academy of Agricultural Sciences, Beijing, 100081, China*.

^2^ *College of Life Science and Engineering, Southwest University of Science and Technology, Mianyang, 621000, China*

**^*^For correspondence:**

E-mail [wangjin@caas.cn](mailto:wangjin@caas.cn); Tel. +86(10)82109868; Fax +86(10)82109868.

**Funding Information**

This study was supported by the Ministry of Agriculture Transgenic Program (No. 2016ZX08009003-002) and National Natural Science Foundation of China (No. 31800061, 31570080, 31500063).


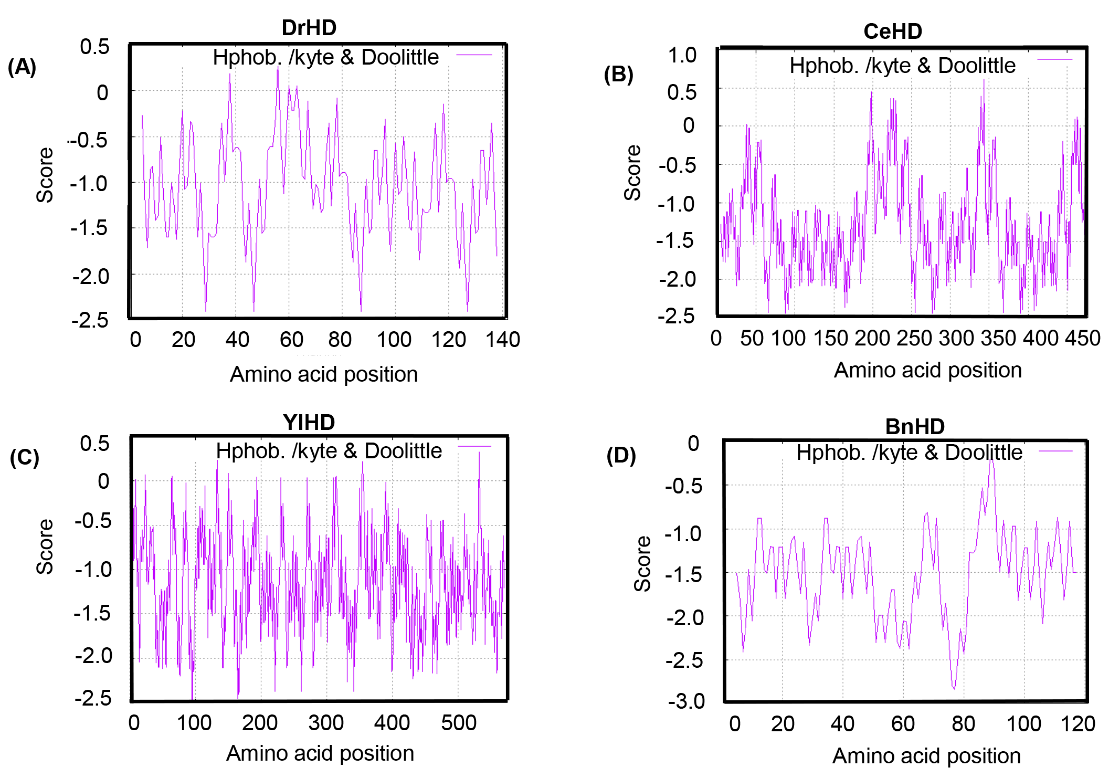


**Fig. S1.** Hydropathic index plot of the HD amino acid sequences analyzed by using the Kyte-Doolittle algorithm. Regions with a hydropathy score below zero are hydrophilic.


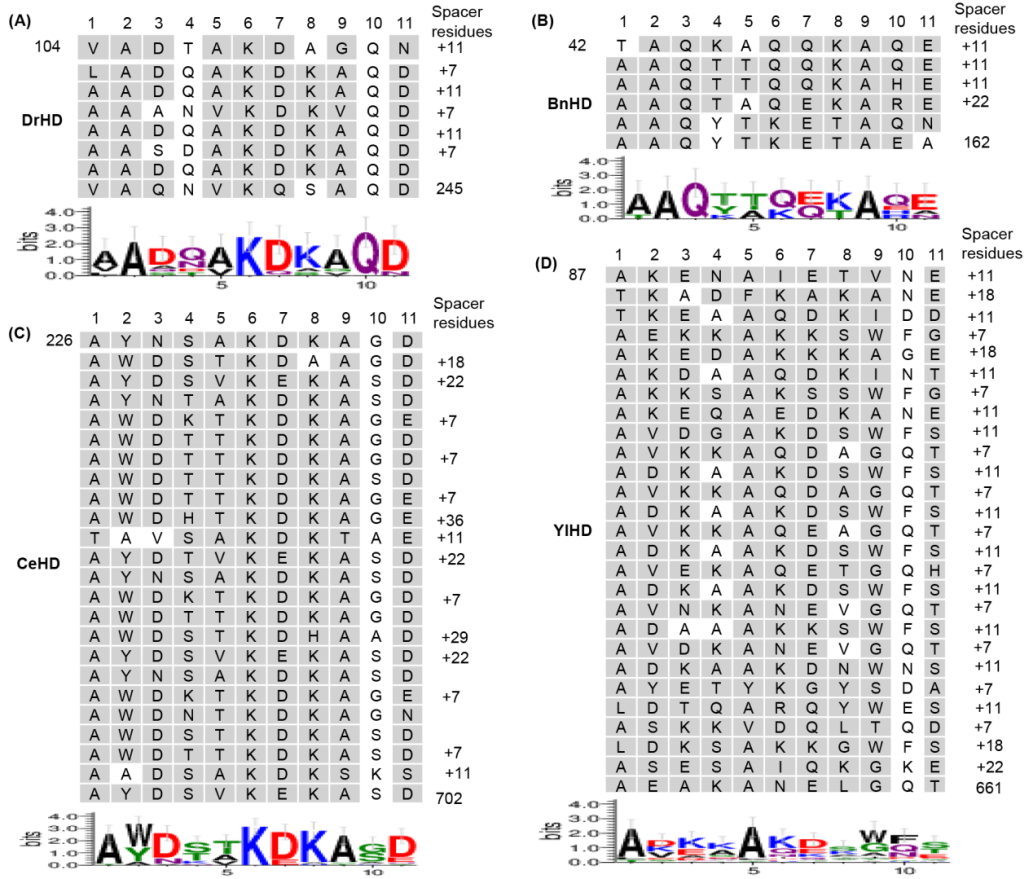


**Fig. S2.** Comparison of the repeating 11-mer motif in HDs.


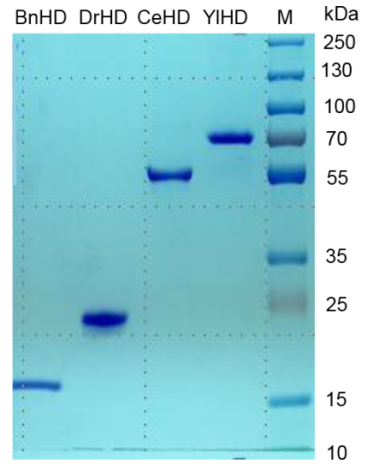


**Fig. S3.** SDS-PAGE analysis of four purified HD proteins. Lane 1, purified BnHD; lane 2, purified DrHD; lane 3, purified CeHD; lane 4, purified YlHD; lane M, molecular weight marker (kDa).


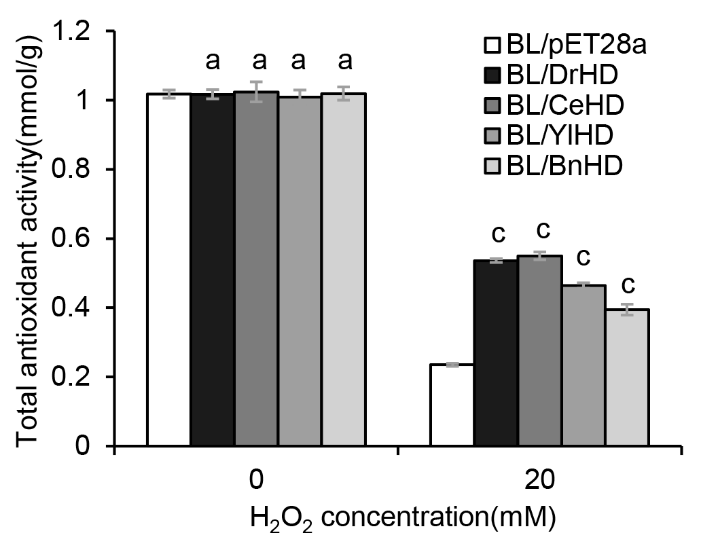


**Fig. S4.** The total antioxidant activity of the transformants was detected by a spectrometer. *E. coli* harbouring the pET28a plasmid served as the control, and this assay was repeated three times (standard deviation shown by error bars). These measurements were performed three times for each case and their *P*-values were calculated based on Tukey multiple comparisons using by R statistics. The letters “a”, “b”, “c” represent “not significantly different (*P* > 0.05)”, “significantly different (0.001< *P* <0.01)” and “extremely significantly different (*P* < 0.001)” respectively compared to LDH alone.


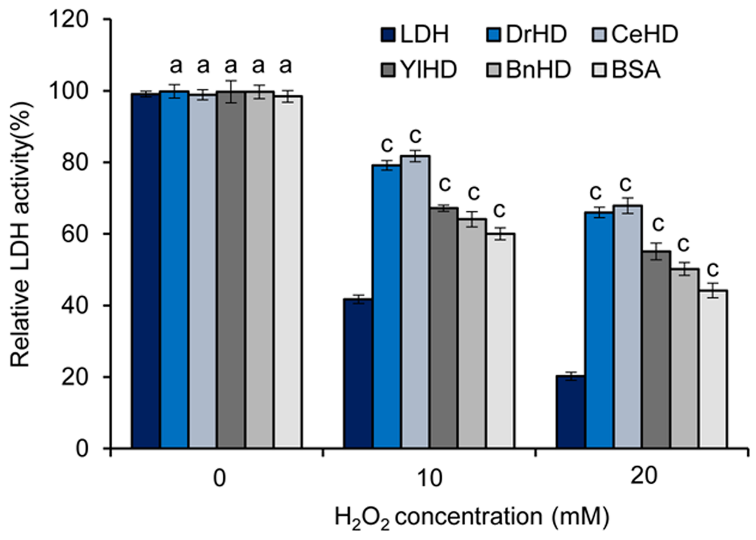


**Fig. S5.** The role of HDs in protecting LDH against H_2_O_2_ stress. The enzymatic activity of LDH (monitored by spectrometer) exposure to H_2_O_2_ with the addition of DrHD, CeHD, YlHD, BnHD and BSA at the mole ratio of 2.5 (HD or BSA):1 (LDH). LDH without additional protectants acted as the control, while BSA was set as the reference for quantitatively specifying HD protection. The activity assay was performed three times for each case. These measurements were performed three times for each case and their *P*-values were calculated based on Tukey multiple comparisons using by R statistics. The letters “a”, “b”, “c” represent “not significantly different (*P* > 0.05)”, “significantly different (0.001< *P* <0.01)” and “extremely significantly different (*P* < 0.001)” respectively compared to LDH alone.


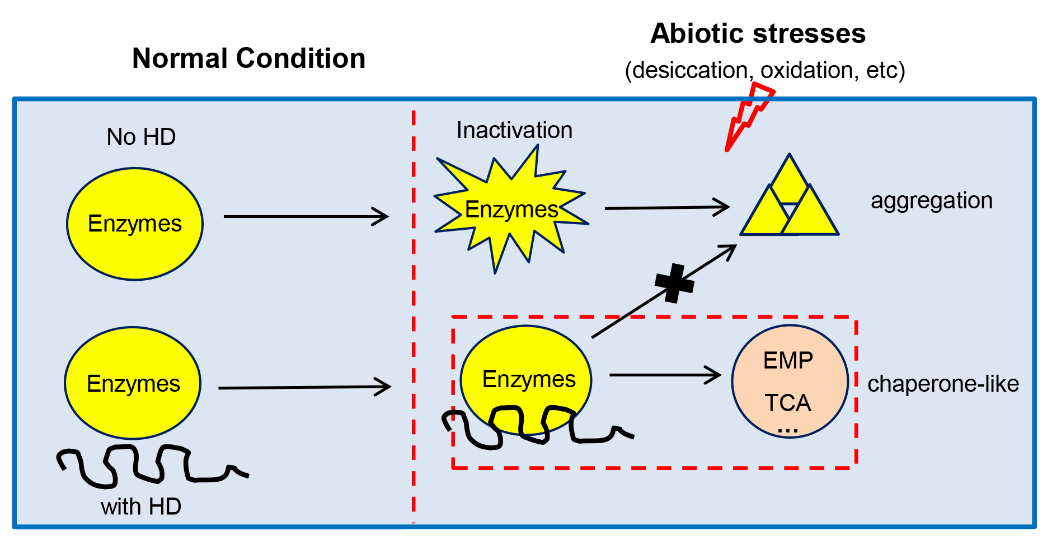


**Fig. S6.** Chaperone-like function model of HD. HD can bind with native proteins, especially crucial enzymes in metabolic pathways, in a weak manner under normal conditions. Their interaction become tight as stresses happened, which leads to the HD’s protection of enzymes from aggregation and accordingly the enzymatic activity was maximumly preserved. In the absence of HD, the enzymes undergo aggregation under stresses and therefore causing inactivation. EMP: Embden-Meyerhof-Parnas pathway; TCA: tricarboxylic acid cycle.

Table S1. List of primers used in this study

| **Gene/Fragment name** | **Primers** | **Sequence (5′–3′)** | **Size (bp)** |
| --- | --- | --- | --- |
| *DrHD* | *DrHD-*F | AGCAAATGGGTCGCGGATCCATGGTGGCCGACACCGCCAAGGA | 472 |
|  | *DrHD-*R | TGGTGGTGGTGGTGCTCGAGTCAGTCCTGAGCGCTTTGCT |  |
| *CeHD* | *CeHD-*F | AGCAAATGGGTCGCGGATCCATGGCTTACAACAGTGCCAAGGA | 1477 |
|  | *CeHD-*R | TGGTGGTGGTGGTGCTCGAGTCAATCAGAAGCT TTTTCCTTAA |  |
| *YlHD* | *YlHD-*F | AGCAAATGGGTCGCGGATCCATGGCCAAGGAGAACGCCATTGA | 1771 |
|  | *YlHD-*R | TGGTGGTGGTGGTGCTCGAGTCAAGTCTGGCCC AGCTCATTTG |  |
| *BnHD* | *BnHD-*F | AGCAAATGGGTCGCGGATCCATGACGGCTCAAAAGGCCCAACA | 409 |
|  | *BnHD-*R | TGGTGGTGGTGGTGCTCGAGTCACGCTTCAGCCGTCTCTTTCG |  |

**Table S2.** Secondary structure content in four HD proteins was obtained by far UV CD spectrometry and calculated with CDPro program

| Treatment | Protein | α-helix (%) | random coil (%) | turn (%) | β-sheet (%) |
| --- | --- | --- | --- | --- | --- |
| Phosphate buffer | DrHD | 12 | 60 | 17 | 11 |
|  | CeHD | 12 | 52 | 17 | 19 |
|  | YlHD | 21 | 41 | 19 | 19 |
|  | BnHD | 15 | 44 | 17 | 24 |
|  | Protein | α-helix (%) | random coil (%) | turn (%) | β-sheet (%) |
| 50%  Glycerol | DrHD | 92 | 1 | 6 | 1 |
|  | CeHD | 90 | 1 | 8 | 1 |
|  | YlHD | 92 | 1 | 6 | 1 |
|  | BnHD | 70 | 5 | 13 | 12 |
|  | Protein | α-helix (%) | random coil (%) | turn (%) | β-sheet (%) |
| 50%  TFE | DrHD | 95 | 1 | 3 | 1 |
|  | CeHD | 95 | 2 | 2 | 1 |
|  | YlHD | 95 | 1 | 3 | 1 |
|  | BnHD | 86 | 2 | 9 | 3 |

**Experimental procedures**

Bioinformatic analysis

The amino acid content and grand average hydropathy (GRAVY) values were estimated by the ProtParam tool (http://web.expasy.org/protparam/). A hydropathy plot was generated with the Kyte and Doolittle algorithm program (<http://web.expasy.org/protscale/>) (Kyte and Doolittle, 1982). Regions of protein disorder were predicted using the CSpritz web server (<http://protein.bio.unipd.it/cspritz/>) (Walsh *et al.*, 2011) and the IUPred (https:// <http://iupred.elte.hu/>) (Dosztányi *et al.*, 2005).

Produced and purification of recombinant proteins DrHD, CeHD, YlHD and BnHD

*Escherichia coli* BL21(DE3) strains carrying recombinant plasmids, named BL/DrHD, BL/CeHD, BL/YlHD and BL/BnHD, were grown in LB medium supplemented with 50 μg ml^-1^ kanamycin at 37°C until OD_600_≈0.6 was reached and then induced with 0.1 mM isopropyl β-D-1-thiogalactopyranoside (IPTG) at 16°C overnight. The cells were harvested, and the pelleted cells were suspended in 50 mM NaH_2_PO_4_, pH 8.0, and 300 mM NaCl. Cells were lysed by sonication on ice and then centrifuged (14 000 *g* at 4°C for 50 min). Affinity chromatography with Ni^+^-NTA Agarose (Cat#R90115; Invitrogen, USA) was used for purification. After washing, the proteins were eluted with elution buffer (50 mM NaH_2_PO_4_, pH 8.0, 300 mM NaCl and 200 mM imidazole). After gel filtration by using an AKTA Pure (GE Healthcare), the proteins were >90% pure as estimated by SDS-PAGE with Coomassie blue staining. Purified proteins were lyophilized and stored at 16°C.

Desiccation and oxidation stress tolerance in *E. coli*

The recombinant *E. coli* strains were grown in LB medium with 50 μg ml^-1^ kanamycin and 0.1 mM IPTG for 4 h at 37°C when the cells reached an OD of 0.6. Cells were then collected by centrifugation to remove the growth medium, washed twice with 10 mM phosphate buffer and resuspended in 1 ml of phosphate buffer. Desiccation stress assays were performed as previously (Rajpurohit and Misra, 2013; Jiang *et al.*, 2017; Wang *et al.*, 2017) with some modification. Briefly, 100 μl of cell suspension was spotted on a sterile glass coverslip, placed inside a sterile petri dish, and incubated in a sealed desiccator maintained at 5% humidity at 30°C. Relative humidity within the desiccator was measured as less than 5% with a hygrometer. The samples were removed at regular intervals (5 and 10 days) and rehydrated by soaking the cell free of the plate in 1 ml of LB medium under sterile conditions for 30 min. For oxidative stress, the cells were added to a final concentration of 20 mM H_2_O_2_ in the dark and incubated for 15 min. Tenfold serial dilutions were made, and 6 μl of each dilution was dripped onto LB agar plates and then incubated at 37°C overnight.

*Measurements of antioxidant capability in E. coli under oxidative stress*

The *E. coli* recombinant cells were induced with 0.1 mM IPTG until OD_600_≈0.6 and then treated with 20 mM H_2_O_2_ or not. Cells were harvested by concentration, washed twice, resuspended in phosphate buffer, and sonicated. The extracts were clarified by centrifugation (14 000 *g* at 4°C for 30 min). The protein concentration was determined by the Bradford method, using bovine serum albumin (BSA) as the standard. The antioxidant activity was measured by an antioxidant capacity kit using the ferric reducing antioxidant power (FRAP) method (Cat#S0116; Beyotime Institute of Biotechnology, China). A working solution was prepared fresh by mixing 2,4,6-tripyridyl-s-triazine (TPTZ) dilution buffer, detection buffer and TPTZ solution in a ratio of 10:1:1(v/v) and then warming it to 37°C. Five microliters of calibration solution, sample or blank was mixed with 180 μl of working solution and kept at 37°C for 5 min. The absorbance of the reaction mixture was recorded at 593 nm. A standard curve was prepared using FeSO_4_ at concentrations ranging from 0.15 to 5 mM. The FRAPs of the samples were calculated from the linear calibration curve and expressed as mmol FeSO_4_ equivalents.

LDH enzymatic activity assay in vitro

Lactate dehydrogenase (LDH, from rabbit, Sigma-Aldrich) activity was used as a marker to test the protective function of additives during abiotic stresses according to previously published methods(Hatanaka *et al.*, 2013; Liu *et al.*, 2013). Purified proteins or BSA were added to equal volumes of enzyme at mass ratios of 2.5:1 (test protein:enzyme). For desiccation treatment, the enzyme mixture was left in a desiccator box for 24 h and then rehydrated in the same volume of 25 mM Tris-HCl (pH 7.5). Samples were then dried, and the rehydration cycle was repeated. For the oxidation treatment, H_2_O_2_ solution was added to the enzyme-protein mixture to obtain final concentrations of 10 and 20 mM, and the samples were incubated for 30 min at 4°C. A change in absorbance (340 nm) was measured after the addition of samples to 600 μl of LDH assay buffer (25 mM Tris-HCl pH 7.5, 100 mM KCl, 2 mM sodium pyruvate and 0.15 mM NADH). LDH activity was monitored as the rate of absorbance change at 340 nm over 1 min due to the conversion of NADH to NAD^+^ at 25°C. The rate determined for the untreated samples was considered 100% in all graphs. All samples were assayed in triplicate, and the corresponding buffer solution without a protectant was used as the blank.

References

Dosztányi, Z., Csizmok, V., Tompa, P., and Simon, I. (2005) IUPred: web server for the prediction of intrinsically unstructured regions of proteins based on estimated energy content. *Bioinformatics* **21:** 3433–3434.

Hatanaka, R., Hagiwara-Komoda, Y., Furuki, T., Kanamori, Y., Fujita, M., Cornette, R., *et al*. (2013) An abundant LEA protein in the anhydrobiotic midge, PvLEA4, acts as a molecular shield by limiting growth of aggregating protein particles. *Insect Biochem. Mol. Biol* **43:** 1055–1067.

Jiang, S., Wang, J., Liu, X., Liu, Y., Guo, C., Zhang, L., *et al*. (2017) DrwH, a novel WHy domain-containing hydrophobic LEA5C protein from *Deinococcus radiodurans*, protects enzymatic activity under oxidative stress. *Sci. Rep* **7:** 1–10.

Kyte, J., and Doolittle, R.F. (1982) A simple method for displaying the hydropathic character of a protein. *J. Mol. Biol* **157:** 105–132.

Liu, Y., Wang, L., Xing, X., Sun, L., Pan, J., Kong, X., *et al*. (2013) ZmLEA3, a multifunctional group 3 LEA protein from maize (*Zea mays* L.), is involved in biotic and abiotic stresses. *Plant Cell Physiol* **54:** 944–959.

Rajpurohit, Y.S., and Misra, H.S. (2013) DR1769, a protein with N-terminal beta propeller repeats and a low-complexity hydrophilic tail, plays a role in desiccation tolerance of *Deinococcus radiodurans*. *J. Bacteriol* **195:** 3888–3896.

Walsh, I., Martin, A.J.M., Di Domenico, T., Vullo, A., Pollastri, G., and Tosatto, S.C.E. (2011) CSpritz: Accurate prediction of protein disorder segments with annotation for homology, secondary structure and linear motifs. *Nucleic Acids Res* **39:** 190–196.

Wang, H., Wu, Y., Yang, X., Guo, X., and Cao, X. (2017) *SmLEA2*, a gene for late embryogenesis abundant protein isolated from *Salvia miltiorrhiza*, confers tolerance to drought and salt stress in *Escherichia coli* and *S. miltiorrhiza*. *Protoplasma* **254:** 685–696.
